# Therapeutic Potential of Propolis in Preclinical Models of Cancer and Infectious Diseases: A Review

**DOI:** 10.3390/ijms26168041

**Published:** 2025-08-20

**Authors:** Michał K. Pierański, Damian Kaniowski, Piotr Szweda

**Affiliations:** 1 Department of Pharmaceutical Technology and Biochemistry, Gdansk University of Technology, Gabriela Narutowicza 11/12, 80-233 Gdansk, Poland; 2 Centre of Molecular and Macromolecular Studies, Polish Academy of Sciences, Sienkiewicza 112, 90-363 Lodz, Poland; damian.kaniowski@cbmm.lodz.pl

**Keywords:** propolis, in vivo, preclinical, anticancer, antibacterial, antifungal, antiparasitic, antiviral

## Abstract

Propolis is a complex, resinous substance originating from plant exudates and processed by bees, e.g., *Apis mellifera* L. Propolis is rich in flavonoids, phenolic acids, and terpenoids. It exhibits broad biological activities, including antimicrobial, anti-inflammatory, immunomodulatory, and anticancer effects. This review summarizes recent findings on the therapeutic potential of propolis in preclinical models of cancer and infectious diseases, with a focus on its molecular mechanisms of action. Experimental data indicate that propolis and its active constituents can induce apoptosis, inhibit proliferation, angiogenesis, and metastasis of cancer cells, and modulate immune responses and microbial virulence. Despite promising in vitro results, in vivo studies remain limited, and their results are often inconsistent. The variability in chemical composition due to geographical and botanical factors, as well as the lack of standardized extracts, further impedes translational research. We highlight key molecular pathways affected by propolis and propose directions for future studies, including improved standardization and more rigorous in vivo results description. These efforts are essential to validate propolis as a potential booster or alternative therapeutic strategy in oncology and infectious diseases treatment.

## 1. Introduction

Propolis, commonly referred to as “bee glue”, is a resinous material processed by honeybees (*Apis mellifera*) from plant exudates mixed with beeswax and salivary enzymes. Bees use it to seal gaps in the hive, stabilize humidity and temperature, and protect the colony from pathogenic microorganisms and predators [1,2]. The term originates from the Greek “pro” (before) and “polis” (city), referring to its defensive function at the hive entrance [3].

Historically, propolis has been valued for its medicinal properties. Although it was used as early as antiquity, interest in its therapeutic applications has resurged in recent decades due to its broad pharmacological potential and the rising demand for natural products in complementary and integrative medicine [4,5].

Chemically, propolis is a highly complex and heterogeneous substance. Its composition varies by botanical source, geography, climate, and season. It contains over 800 identified compounds, including flavonoids, phenolic acids, terpenoids, aromatic acids, steroids, and essential oils [6,7,8,9]. The gross components of propolis are: 50% resin and vegetable balsam, 30% wax, 10% essential and aromatic oils, 5% pollen and 5% various other substances, including organic debris [10]. Among the most studied types are poplar propolis (rich in flavones and phenolic esters from *Populus* spp.), Brazilian green propolis (notably containing artepillin C from *Baccharis dracunculifolia*), and red propolis (rich in isoflavonoids derived from *Dalbergia ecastaphyllum*) [11,12,13].

Propolis exhibits a wide range of biological activities supported by numerous in vitro and in vivo studies, including antioxidant [14], anti-inflammatory [15], antimicrobial [16], antiviral [17], immunomodulatory [18], and anticancer properties [19]. These effects are attributed to its bioactive constituents, such as caffeic acid phenethyl ester (CAPE), chrysin, galangin, kaempferol, and pinocembrin, which modulate various cellular signalling pathways associated with apoptosis, inflammation, and cell cycle regulation [20,21,22].

Although substantial evidence supports the pharmacological potential of propolis, most studies have been conducted in vitro. In contrast, in vivo studies remain limited, and clinical trials are rare. The few explored clinical applications include the use of propolis in oral care, wound healing, and as an adjunctive therapy in respiratory infections, including COVID-19 [23,24,25]. The wide chemical variability, lack of standardized extracts, and limited pharmacokinetic data pose major challenges to its broader therapeutic implementation [26,27,28].

Given the global rise of drug-resistant infections and the continued need for more effective and less toxic anticancer therapies, natural products such as propolis offer promising but underexplored possibilities for drug discovery. This review summarizes current evidence on the therapeutic applications of propolis in animal models of cancer and infectious diseases. Moreover, it highlights key gaps in preclinical research that must be emphasized to advance its development as a therapeutic agent.

## 2. Effect of Propolis Monotherapy on Tumor Xenograft Models

### 2.1. Breast Cancer

Breast cancer remains a leading cause of mortality among women, with growing evidence implicating cardiovascular complications as a significant contributor to patient deaths. Ehrlich ascites carcinoma (EAC), a spontaneously arising murine mammary adenocarcinoma, is widely utilized to investigate tumor pathogenesis and evaluate anticancer agents. EAC is known to metastasize to multiple organs, including the lungs, liver, and bones. Clinical observations also link cancer progression to cardiac alterations at both morphological and molecular levels, affecting cardiac function [29].

In a study involving Swiss mice, EAC cells were implanted, and the mice were treated with Egyptian propolis (50 mg/kg, intraperitoneally (i.p.) for 11 days), starting two hours prior to tumor cells implantation. The treatment significantly reduced tumor volume and increased the number of dead tumor cells. This was accompanied by prolonged mean survival time, an improved lifespan index, and a higher treated-to-control ratio. Immunological analyses revealed a marked enhancement in lymphocyte transformation, phagocytic capacity, and cytotoxic activity. Additionally, propolis treatment improved hematological and biochemical parameters, including increased red blood cell count, decreased leukocytosis, reduced creatinine levels, and elevated total globulin and albumin levels compared to control [30].

The antitumor activity of Egyptian propolis (4 mg/kg/day, intraperitoneally for nine days) was demonstrated in a breast cancer model, highlighting its influence on multiple molecular mechanisms. These included upregulation of antioxidant defenses, inhibition of angiogenesis, suppression of inflammatory and mTOR signaling pathways, and induction of apoptosis. Importantly, the treatment led to increased expression of miRNA-223 in tumor tissues, which may contribute to the deregulation of key cell cycle checkpoints [31,32].

The therapeutic efficacy of propolis was further amplified when formulated into nanostructured lipid carriers (ProE-NLC), administered at the same dosage and schedule. This formulation enabled targeted delivery to breast tumors and maintained physical stability for up to nine months, with only minimal increases in particle size due to fusion. These results suggest that ProE-NLC holds promise as a novel therapeutic platform for breast cancer [32]. Taken together, these studies indicate that propolis consistently reduces tumor growth and enhances survival in breast cancer models, primarily through apoptosis induction and immune activation.

### 2.2. Cervical Cancer

Cervical cancer is the fourth most prevalent cancer among women worldwide, with the highest incidence occurring in sub-Saharan Africa, Central America, and Southeast Asia. These regional disparities are primarily attributed to limited access to human papillomavirus (HPV) vaccination, inadequate screening and treatment programs, higher prevalence of human immunodeficiency virus (HIV), and socioeconomic challenges. HPV, a common sexually transmitted infection, is the principal etiological agent of cervical cancer. However, women living with HIV are six times more likely to develop this cancer. While progression from HPV infection to cervical cancer typically spans 15–20 years, this timeline may be shortened to 5–10 years in immunocompromised individuals, particularly those with untreated HIV. Standard treatment usually begins with surgical intervention, followed by systemic therapies as clinically indicated [33].

The therapeutic effects of propolis granules derived from northern Thailand (24 mg/kg, orally once daily) were assessed in female CB-17 SCID mice inoculated subcutaneously with HeLa cancer cells in the right mammary fat pad. The propolis-treated group exhibited increased apoptosis, confirmed by strong DNA fragmentation as demonstrated by TUNEL assay. Histopathological evaluation with hematoxylin and eosin (H&E) staining revealed minor morphological changes in the treated tumors compared to controls. Moreover, the survival time of the treated group was significantly extended—doubling to 60 days—relative to the PBS-treated control. These findings suggest that propolis may serve as a valuable natural adjunctive or complementary therapeutic option in cervical cancer management [34]. Overall, the evidence suggests that propolis exerts marked cytotoxic and pro-apoptotic effects in cervical cancer models, highlighting its potential as a alternative therapeutic option.

### 2.3. Colorectal and Gastrointestinal Cancer

Gastrointestinal (GI) cancers encompass a broad group of malignancies affecting the digestive tract, with the most common sites being the esophagus, stomach, colon, liver, and pancreas. Despite recent advancements in diagnostic tools and treatment modalities, significant challenges remain in achieving long-term disease control [35].

In a human colorectal cancer xenograft, oral administration of Iraqi propolis at doses of 500 mg/kg and 1000 mg/kg for 21 consecutive days significantly inhibited tumor growth derived from HCT-116 cells. Tumor weight was reduced by approximately 60% in the treated group compared to controls. Histological evaluation revealed extensive degeneration of cancer cells in treated tumors, along with ulceration, interstitial fibrosis, and marked inflammatory cell infiltration. Furthermore, expression of the tumor suppressor gene p53 increased to 40.8% in treated animals, compared to 16% in controls. Expression of the proliferation marker (Ki-67) was notably reduced in propolis-treated tumors [36].

To further support the therapeutic value of propolis in gastrointestinal cancer, Philippine propolis was evaluated in A4gnt knockout mice, which serve as a robust model for differentiated-type gastric adenocarcinoma. Oral administration of Philippine propolis (100 mg/kg daily) for 30 days resulted in significant regression of gastric mucosal elevations. Histological examination showed a substantial reduction in pyloric mucosal thickness and T-lymphocyte infiltration, indicating tumor suppression and immune modulation in gastric cancer [37].

The effects of propolis (300 mg/kg, intraperitoneally, daily for 14 days) were investigated in a syngeneic CT26 tumor-bearing mouse model under immunosuppressed conditions. Treatment significantly reduced both tumor size and weight, likely mediated by enhanced apoptosis and inhibition of angiogenesis. Immunological assessments showed increased proportions of CD3⁺ and CD4⁺ T cells and an elevated CD4⁺/CD8⁺ ratio. The antitumor effects were linked to activation of the TLR4–MAPK signaling pathway, which enhanced macrophage-mediated immune responses and promoted the secretion of key pro-inflammatory cytokines, including IL-1β, IL-6, and TNF-α [38]. Collectively, findings in gastrointestinal tumor models point to strong antiproliferative and anti-angiogenic effects of propolis, often mediated by NF-κB inhibition and modulation of immune responses.

### 2.4. Epidermoid Carcinoma

The epidermoid carcinoma cell line (A431) is extensively employed in preclinical studies to investigate novel anticancer agents, particularly those targeting the epidermal growth factor receptor (EGFR). In normal cells, EGFR expression typically ranges between 4 × 10^4^ and 1 × 10^5^ receptors per cell. However, A431 cells exhibit EGFR overexpression, with up to 2 × 10^6^ receptors per cell, making them a valuable model for EGFR-targeted therapies [39].

Due to this characteristic, A431 cells serve as a valuable model for EGFR-mediated delivery of therapeutic agents, including nucleic acids modified with boron clusters [40,41,42], monoclonal antibodies [43], or tyrosine kinase inhibitors [44].

Given this model’s relevance, Poplar propolis was evaluated in BALB/c nude mice bearing A431 xenograft tumors. Mice were treated with intragastric gavage of propolis at doses of 50 or 100 mg/kg daily for 12 consecutive days. A significant reduction in tumor volume was observed in the 100 mg/kg treatment group compared to PBS controls, indicating a potent in vivo antitumor effect. Conversely, the 50 mg/kg dose did not yield a statistically significant reduction. Histopathological analysis using H&E staining confirmed these findings, revealing extensive tumor cell necrosis in the high-dose group, consistent with a cytotoxic mechanism of action [45].

### 2.5. Fibrosarcoma

Adult fibrosarcoma is a rare and aggressive soft tissue cancer, predominantly affecting middle-aged and older adults, with a slight male predominance. It typically originates in the deep tissues of the extremities, trunk, head, or neck. Arising from mesenchymal cells, fibrosarcomas are characterized by the uncontrolled proliferation of spindle-shaped fibroblasts. The recurrence rate is approximately 50%, and the tumors exhibit a poor response to conventional chemotherapy, likely due to inherent resistance mechanisms [46].

To explore alternative treatment strategies, the antitumor activity of Brazilian green propolis extract (GPSE) and its γ-cyclodextrin inclusion complex (GPSE-γCD) was assessed using a human HT-1080 fibrosarcoma xenograft model. Mice were orally administered 100 mg/kg of GPSE or GPSE-γCD every other day for three weeks. Tumor growth was reduced by ~30% in the GPSE group and ~50% in the GPSE-γCD group compared to PBS-treated controls. Notably, no adverse effects on body weight or physical activity were observed, indicating favorable tolerability. These findings support the potential of GPSE-γCD as a more potent and well-tolerated natural antitumor agent for fibrosarcoma. However, only modest effects on fibrosarcoma growth in vivo suggest model and composition-dependent variability and require further investigation [47].

### 2.6. Hodgkin Lymphoma

Hodgkin lymphoma is a B-cell-derived malignancy defined by the presence of a small number of malignant cells within a heterogeneous tumor microenvironment rich in immune effector cells. It most commonly affects adolescents and young adults, although cases are also observed in older populations. Diagnosis relies on histopathological and immunohistochemical evaluation of lymph node biopsies. The therapeutic goal is to achieve long-lasting remission while minimizing long-term toxicities associated with treatment [48].

In a preclinical study, Dalton’s lymphoma ascites cells were intraperitoneally inoculated into Swiss albino mice. These tumor-bearing mice were then treated with Indian propolis at doses ranging from 100 to 400 mg/kg (i.p.) over 14 days. Propolis treatment significantly increased the average lifespan of tumor-bearing mice. In the group treated with 400 mg/mL of propolis 90% of mice reached the average lifespan, compared to 48% in the untreated control and 96% in the 5-fluorouracil (5-FU) monotherapy group. Additionally, the proportion of viable tumor cells was significantly reduced in the propolis-treated group relative to the PBS control.

Haematological assessments revealed normalization of haemoglobin concentration, red blood cell (RBC) count, and white blood cell (WBC) count, indicating restoration of hematopoietic function. Biochemical analyses further demonstrated that propolis treatment normalized serum levels of total cholesterol, triglycerides, aspartate aminotransferase (AST), alanine aminotransferase (ALT), and alkaline phosphatase (ALP), suggesting hepatoprotective effects. Collectively, these findings highlight the dual benefits of Indian propolis in exerting antitumor effects and mitigating systemic toxicity, supporting its potential role as an adjunctive therapy in Hodgkin lymphoma [49]. Taken together, preclinical evidence indicates that propolis exerts anticancer activity across diverse tumor types through convergent mechanisms: apoptosis induction, inhibition of proliferation and angiogenesis, immune modulation, and regulation of NF-κB and mTOR signaling. Nonetheless, variability in outcomes across models highlights the importance of the extraction method, composition, and dosing strategy.

## 3. Preclinical Evaluation of Propolis-Based Combination Therapies in Cancer Treatment

### 3.1. Methotrexate

Methotrexate (MTX) is an antimetabolite chemotherapeutic agent that exerts its anticancer activity primarily by inhibiting dihydrofolate reductase (DHFR), thereby reducing tetrahydrofolate availability and impairing DNA synthesis in rapidly proliferating cells. The combined effects of MTX and Egyptian propolis were evaluated in a murine Ehrlich ascites carcinoma (EAC) model.

Mice received oral Egyptian propolis (500 mg/kg) for two days prior to intraperitoneal (i.p.) inoculation with EAC cells. Post-implantation, daily oral administration of propolis (500 mg/kg) continued for two weeks, either alone or in combination with MTX (2.5 mg/kg, i.p., administered every other day). The combination therapy led to a synergistic tumor-inhibitory effect, reducing tumor volume by approximately 82%, compared to a 50% reduction achieved with monotherapy. The data suggest that propolis enhances tumor cell sensitivity to MTX, likely via its antioxidant and immunomodulatory properties [50].

Additionally, propolis mitigated MTX-induced hepatotoxicity and nephrotoxicity. Biochemical markers of liver and kidney injury, elevated in the MTX-only group, were significantly lower in groups receiving propolis, either alone or in combination. DHFR expression was lowest in the combination treatment group, correlating with the highest levels of DNA damage in EAC cells. These findings underscore the potential of Egyptian propolis as a therapeutic adjuvant that enhances the efficacy of MTX while reducing its systemic toxicity [50].

### 3.2. Fluorouracil

Fluorouracil (5-fluorouracil, 5-FU) is a widely used chemotherapeutic agent approved by the U.S. Food and Drug Administration for the treatment of gastric, pancreatic, breast, and colorectal adenocarcinomas. It functions primarily by inhibiting thymidylate synthase, thus interfering with DNA synthesis. Additionally, 5-FU is incorporated into RNA and DNA in cancer cells, disrupting their function and ultimately leading to apoptosis in proliferating tumors [51].

The synergistic potential of propolis and 5-FU has been evaluated in several in vivo models:

- In a BALB/c mouse model of chemically induced colorectal cancer, co-administration of Iranian propolis (90 mg/kg, i.p., five times per week) and 5-FU (50 mg/kg, i.p., once weekly) for eight weeks significantly reduced tumor burden. Histopathological evaluation showed reduced β-catenin, COX-2, and iNOS expression in the combination therapy group compared to monotherapy groups, suggesting improved chemotherapeutic efficacy and inflammation control [52].
- In a LoVo colorectal cancer xenograft model, the combination of Anatolian propolis extract (400 mg/kg, i.p.) with 5-FU (10 mg/kg, i.p.), administered daily for three weeks, demonstrated superior tumor inhibition relative to either agent alone. Intraperitoneal administration of propolis produced better outcomes than oral administration. Notably, serum levels of pro-inflammatory cytokines (TNF-α, IL-1, IL-6) were reduced in the combination and propolis-alone groups. Additionally, the activity of liver enzymes: ALT (alanine aminotransferase) and AST (aspartate aminotransferase) was significantly lower, indicating reduced hepatotoxicity [53].
- A separate study explored sex-dependent effects of propolis combined with 5-FU. Mice pretreated with crude Croatian propolis powder (300 mg/kg in the diet) for 14 days prior to 4T1 mammary carcinoma inoculation received 5-FU (200 mg/kg, i.p.) one day post-injection. In male mice, the combination significantly reduced tumor growth and metastatic spread, a benefit not observed in female mice. This sex-specific difference may be attributed to lower dihydropyrimidine dehydrogenase (DPD) activity in males, resulting in higher 5-FU sensitivity [54].

Further advancements include propolis-loaded nanostructured lipid carriers (ProE-NLC; 4 mg/kg/day, i.p., for 9 days) used in EAC-bearing mice. Compared to conventional propolis, ProE-NLC exhibited enhanced antitumor efficacy by modulating multiple pathways: increasing antioxidant capacity, inhibiting angiogenesis and inflammation, suppressing mTOR signaling, and inducing apoptosis. These effects were linked to increased tumor expression of miRNA-223. When ProE-NLC (4 mg/kg/day) was co-administered with 5-FU (20 mg/kg/day, i.p., for 9 days), the combination exhibited potent synergistic activity and reduced toxicity, suggesting its potential as both a monotherapy and adjuvant in breast cancer treatment [32].

A novel nanoparticle formulation combining sericin, propolis, and 5-FU (SER/PRO/5-FU), was administered at 75 mg/kg via gastric gavage for eight weeks in a colorectal carcinoma model. The SER/PRO/5-FU complex exhibited the strongest synergistic effect among treatment groups. Mechanistically, it inhibited the PI3K/AKT/mTOR pathway while activating FOXO-1, leading to enhanced autophagy, apoptosis, and decreased reactive oxygen species (ROS) levels. Histological findings confirmed both preventive and therapeutic efficacy [55].

### 3.3. Irinotecan

Irinotecan (IRI) is a topoisomerase I inhibitor widely used in the treatment of advanced colorectal cancer and other solid tumors, including non-small cell lung, pancreatic, biliary tract, gastric, and cervical cancers. Although it can be administered as monotherapy, irinotecan is more frequently used in combination regimens with agents such as 5-fluorouracil, oxaliplatin, or targeted monoclonal antibodies (e.g., cetuximab, bevacizumab). More recently, experimental models have explored combinations with natural compounds like propolis to enhance therapeutic efficacy [56].

To evaluate the synergistic effects of irinotecan and propolis, Swiss albino mice were engrafted with Ehrlich ascites tumor (EAT) cells. Propolis was administered at 100 mg/kg daily for three consecutive days starting on day 3 after tumor cell inoculation. Irinotecan (50 mg/kg, i.p.) was administered on days 1, 13, and 19. Combination therapy significantly reduced the tumor volume in the peritoneal cavity compared to untreated EAT-bearing mice.

Moreover, the proportion of neutrophils in the peritoneal fluid was significantly elevated in the combination treatment group. Given that ROS generation by neutrophils and macrophages is closely tied to their functional activity, and that propolis possesses notable antioxidant properties, these findings suggest that propolis may modulate immune responses and cell survival. Overall, co-administration of propolis with irinotecan enhanced antitumor efficacy and prolonged survival in tumor-bearing mice, indicating the need for further mechanistic investigation of this herb–drug synergy [57].

### 3.4. Dual Model: Cancer and Candida Albicans Infection

*C. albicans* is a common opportunistic fungal pathogen that poses a serious threat in immunocompromised patients, including those undergoing cancer treatment. The host response to *C. albicans* involves both innate and adaptive immunity, including neutrophils, macrophages, and cytokines that regulate inflammation and T-cell differentiation.

A study investigated the immunomodulatory effects of propolis (100 mg/kg, oral gavage for 10 days) in mammary tumor-bearing mice co-infected with *C. albicans*. Propolis treatment led to approximately 47% reductions in interleukin-4 (IL-4) and interleukin-10 (IL-10), indicating suppression of anti-inflammatory cytokine signaling. In contrast, transforming growth factor-beta (TGF-β), tumor necrosis factor-alpha (TNF-α), and interferon-gamma (IFN-γ) levels were significantly elevated, pointing to enhanced pro-inflammatory and antitumor responses.

Interestingly, IL-17 levels, associated with Th17-driven antifungal defense, were reduced, suggesting a regulatory effect on inflammatory balance. Collectively, these results indicate that propolis exerts both antifungal and antitumor effects by modulating immune responses and enhancing the host’s ability to combat malignancy and infection simultaneously [58].

Further supportive evidence comes from a study evaluating the combined effects of propolis (66 mg/kg/day, oral gavage for 14 days) and acidophilus milk in a 4T1 breast cancer murine model. The combination therapy achieved the highest tumor inhibition rate (63.39%) compared to either treatment alone. Additionally, the combination enhanced splenocyte proliferation in response to mitogens such as concanavalin A (ConA), lipopolysaccharide (LPS), and phytohemagglutinin (PHA), indicating robust immunostimulation. Elevated IFN-γ production further supported the synergistic antitumor and immunomodulatory effects of this combination therapy, suggesting its potential utility as an adjunct in breast cancer management [59].

### 3.5. Photodynamic Therapy

Photodynamic therapy (PDT) is an FDA-approved, minimally invasive treatment modality for certain cancers and non-malignant conditions. It involves the administration of a photosensitizing agent followed by localized irradiation with light at a specific wavelength corresponding to the agent’s absorption spectrum. This process generates ROS that induce apoptosis or necrosis in malignant cells while sparing surrounding healthy tissue [60].

In a preclinical study, BALB/c-nu mice were xenografted with A431 cancer cells. Tumors were pre-treated with Brazilian green propolis (BGP) extract prior to exposure to a single dose of PDT. Xenograft excised six days post-treatment exhibited a significantly enhanced apoptotic activity in the BGP-PDT combination group.

Mechanistically, BGP treatment reduced the expression of the anti-apoptotic protein Bcl-xL and upregulated key pro-apoptotic markers, including Bax, NOXA, and cleaved caspase-3, indicating activation of the intrinsic mitochondrial apoptotic pathway. Furthermore, BGP suppressed PDT-induced activation of inflammatory and survival signalling via the p-IKK and NF-κB pathways.

These findings suggest that BGP acts as a sensitizing agent, augmenting the efficacy of PDT by enhancing pro-apoptotic signalling and attenuating cellular survival and inflammatory pathways. Thus, Brazilian green propolis may be a promising natural adjuvant for improving PDT outcomes in skin and other EGFR-driven tumors [61]. Most of the publications related to anti-cancer and synergistic activity of propolis are summarized in Table 1.

## 4. Adjuvant Use of Propolis in Oncology: Mitigating Treatment-Related Side Effects

A significant limitation of chemotherapy is its non-selective toxicity to healthy tissues, often leading to organ damage, hematological disorders, and impaired quality of life. Increasingly, natural products such as propolis are being investigated for their protective effects against treatment-induced toxicities due to their antioxidant, anti-inflammatory, and immunomodulatory properties.

### 4.1. 5-Fluorouracil-Induced Cardiotoxicity

Administration of 5-fluorouracil (5-FU) at 125 mg/kg/day (i.p.) for 14 days in rats resulted in pronounced cardiotoxicity, evidenced by elevated malondialdehyde (MDA), increased expression of cyclooxygenase-2 (COX-2) and tumor necrosis factor-alpha (TNF-α), and histopathological degeneration in cardiac tissue. Haematological disturbances, reduced antioxidant defences (TAC, catalase), and weight loss were also observed.

Co-treatment with ethanolic extract of propolis (250 mg/kg/day, oral gavage) significantly reversed these side effects. Propolis reduced COX-2 expression and inflammatory markers, restored antioxidant status, and normalized blood parameters, indicating cardioprotective and anti-inflammatory efficacy in 5-FU-treated rats [62].

### 4.2. Mitomycin C-Induced Toxicity

Mitomycin C (MMC) is a potent DNA crosslinking agent used in treating various solid tumors. However, it is associated with significant genotoxic and cytotoxic side effects.

In Swiss albino mice, pretreatment with Indian propolis (400 mg/kg, i.p.) one hour before MMC (8 mg/kg, i.p.) significantly mitigated MMC-induced bone marrow suppression and systemic toxicity. The protective effects were attributed to the antioxidant, free radical scavenging, and lipid peroxidation-inhibitory properties of propolis [63].

Further investigations showed that MMC at doses of 2, 4, and 8 mg/kg induced dose-dependent testicular toxicity by day 35 post-treatment. Pretreatment with propolis significantly improved testicular function and reduced DNA damage markers (γ-H2AX, RAD51, KU80), while restoring testosterone and inhibin B levels. These data support the genoprotective and endocrine-restorative effects of propolis in MMC-induced reproductive toxicity [64].

### 4.3. Protection Against Common Chemotherapeutic Agents

Doxorubicin, cisplatin, irinotecan, and cyclophosphamide are widely used in oncology but are associated with serious adverse effects, including myelosuppression, nephrotoxicity, hepatotoxicity, and reproductive toxicity [65,66]. Propolis has shown protective effects in multiple models:

#### 4.3.1. Cisplatin

Propolis (50–100 mg/kg, i.p.) enhanced the antitumor activity of cisplatin while reducing toxicity in Ehrlich ascites tumor-bearing mice. Combination treatment increased survival by 160.3% compared to propolis monotherapy [67]. In a nephrotoxicity model, propolis (100 mg/kg, i.p., for 14 days) significantly reduced cisplatin (7 mg/kg)-induced renal damage, preserving brush border integrity and reducing histopathological alterations [68].

#### 4.3.2. Irinotecan

In mice treated with irinotecan (50 mg/kg, i.p., for 5 days), pre-treatment with propolis (100 mg/kg) or related flavonoids (naringin, quercetin) resulted in reduced peritoneal cell counts, enhanced macrophage and PMN activity, protection of hepatic and renal tissues, and reduced frequency of micronucleated cells. According to the authors, these changes were associated with immune activation and tumor suppression [69].

#### 4.3.3. Doxorubicin

Egyptian propolis (200 mg/kg, orally, 5 days/week) effectively prevented doxorubicin (3 mg/kg, i.p.)-induced testicular damage by restoring steroidogenic gene expression (3β-HSD, 17β-HSD, StAR) and reducing oxidative stress and apoptosis [70], Similarly, propolis (250 mg/kg, i.p.) given 14 days before doxorubicin (25 mg/kg, i.p.) improved liver function and oxidative stress parameters [71].

#### 4.3.4. Cyclophosphamide

Cyclophosphamide (CTX; 200 mg/kg, i.p., for 12 days) causes elevations in serum ALT, AST, urea, and creatinine, along with WBC and platelet depletion and structural damage to liver and kidney tissues. Co-administration of propolis (100 mg/kg, i.p.) improved biochemical markers and restored hematologic parameters, demonstrating protective efficacy [72].

### 4.4. Radiotherapy-Induced Toxicity

While radiotherapy (RT) is a cornerstone of cancer treatment, it induces inflammation and vascular remodelling, contributing to tissue damage and tumor recurrence [73]

Several studies support the antiangiogenic and tissue-protective effects of propolis:

- In a 40-week rat model of bladder cancer, propolis (300 mg/kg/day, i.p.) significantly inhibited microvascular density, indicating suppression of tumor-induced angiogenesis [74].
- Brazilian green propolis (300 mg/kg, s.c., for 5 days) reduced retinal neovascularization in a murine retinopathy model without impairing physiological revascularization [75].
- Brazilian red propolis (200 mg/kg, oral gavage for 11 days) also inhibited angiogenesis in a hamster cheek pouch tumor model [76].

**Figure 1:**
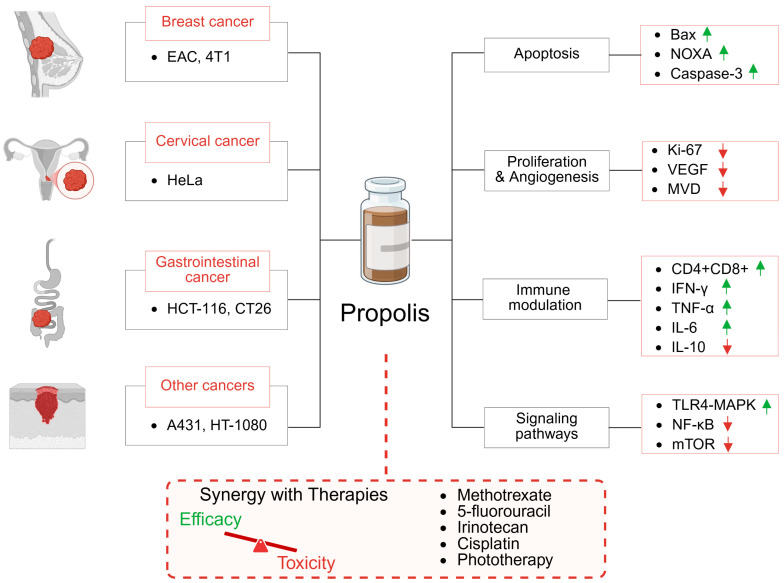
Anticancer effects and synergy with existing therapies in in vivo models. The green arrows indicate upregulation, and the red arrows indicate downregulation of the signaling molecules.

Anatolian propolis (200 mg/kg) mitigated radiotherapy-induced mandibular osteoradionecrosis (ORN) in a rat model exposed to 35 Gy. Although the study lacked molecular endpoints such as ELISA or RT-PCR, it showed increased expression of osteogenic markers (BMP-2, TGF-β3), indicating bone regenerative potential [77].Furthermore, intravenous administration of propolis (200 mg/kg, for 14 days) effectively reduced radiation-induced oral mucositis and prevented the development of radio-resistance by alleviating tumor hypoxia [78]. However, in another study, although propolis (400 mg/kg, i.p., for 70 days) preserved salivary gland function, it did not prevent gamma radiation-induced tissue damage [79]. Most of the publications related to toxicity reducing properties of propolis are summarized in Table 2. In addition, Figure 1 schematically illustrates the potential anticancer applications of propolis together with its possible mechanisms of action. In summary, propolis demonstrates activity against various tumor models (breast, cervical, gastrointestinal, and other cancers) by inducing apoptosis, inhibiting proliferation and angiogenesis, modulating immune responses, and regulating signaling pathways. It also enhances the efficacy and reduces the toxicity of conventional anticancer therapies.

## 5. Antiparasitic Effect of Propolis

Parasitic infections such as toxoplasmosis, leishmaniasis, schistosomiasis, and other protozoan and helminthic diseases represent a major global health burden, particularly in resource-limited regions. *Toxoplasma gondii* infects an estimated one-quarter of the human population worldwide, with the highest rates (>60%) observed in Africa and South America. Infection poses significant risks for immunocompromised individuals and pregnant women, often leading to congenital toxoplasmosis or severe cerebral disease in newborns [80].

Leishmaniasis, including its cutaneous and visceral forms, affects approximately 6 million people across nearly 100 countries, with up to 1.6 million new cases and tens of thousands of deaths annually [81]. Meanwhile, *Schistosoma mansoni* infection contributes to the most pervasive parasitic disease globally, with over 250 million individuals infected and roughly 130,000 deaths per year, predominantly in sub-Saharan Africa [82].

The clinical challenges posed by these pathogens—including high morbidity, mortality, drug resistance, and adverse effects of current medications—underscore the urgent need for novel, effective, and safe therapeutic agents [83]. The following section reviews experimental evidence demonstrating the antiparasitic activity of propolis in various in vivo models of *T. gondii*, *Leishmania* spp., *Schistosoma mansoni*, *Trypanosoma* spp., *Giardia lamblia*, and related pathogens.

### 5.1. Toxoplasma Gondii

In the first study mice were infected intraperitoneally with *T. gondii*. An ethanolic extract of Egyptian propolis was administered orally at a dose of 150 mg/kg/day for 7 days. Propolis was also loaded into chitosan/alginate nanoparticles, with or without the addition of spiramycin. While propolis alone demonstrated modest antiparasitic activity (40.2–59.6% reduction in parasite burden) and extended survival by two days compared to untreated controls, its efficacy was considerably enhanced when incorporated into nanoparticle formulations, particularly when combined with spiramycin. The synergistic propolis/spiramycin formulation extended survival by 14 days and reduced parasite burden in the liver, spleen, and brain by 96–98.8%. The authors emphasized the nanoparticles’ ability to penetrate tissues and cross the blood–brain barrier (BBB), suggesting a promising platform for treating acute toxoplasmosis [84].

A series of studies by Ehab Kotb Elmahallawy and collaborators assessed the efficacy of propolis and wheat germ oil, individually and in combination, against acute toxoplasmosis in Swiss albino mice. In the first two studies, 0.1 mL of Egyptian propolis extract was administered orally for 10 consecutive days, starting six weeks post-infection. Propolis reduced the mean number of liver tissue cysts and ameliorated histopathological changes in liver and lung tissues, although the combination with wheat germ oil showed greater therapeutic efficacy, including complete restoration of histological architecture [85]. The second study demonstrated a significant reduction in brain parasite load following propolis treatment, along with improved histopathology in the brain, uterus, and kidneys. The effect was more pronounced in the propolis and wheat germ oil combination group, especially when parasite burden was measured by real-time PCR [86].

In the two following studies by the same group, the treatment regimen (0.1 mL/day of propolis for 10 days) began one day after infection. In one study, propolis, alone and in combination, significantly reduced liver parasite burden, as confirmed by both stained tissue smears and qRT-PCR. Histopathological examination revealed reduced mononuclear infiltration, hepatocellular necrosis, and peritoneal oedema. The spleens of treated mice showed normalized white and red pulp size and decreased megakaryocyte numbers [87]. In the final study of the series, both treatments significantly reduced brain parasite load, as assessed by cyst counting and qRT-PCR. Propolis also helped restore tissue architecture in the brain, uterus, and kidneys, as reflected by improved total lesion scores [88].

### 5.2. Leishmania spp.

In a study involving mice infected subcutaneously with *L. amazonensis*, treatment with a gel containing 2.5% Brazilian red propolis—alone or in combination with Glucantime drug—was evaluated. Propolis alone was not effective in reducing lesion development or size. However, when combined with Glucantime, it resulted in less exudative lesions and higher interferon-gamma (IFN-γ) levels, indicating an enhanced immune response [89].

In another model of intradermal *L. amazonensis* infection in the murine ear dermis, daily topical application of a gel containing 3.6% Brazilian green propolis glycolic extract was initiated three weeks post-infection and continued for up to 12 weeks. Although parasite load in ear and lymph nodes was unaffected, lesion size was reduced in treated animals. When combined with Glucantime, lesion size was further reduced. The authors concluded that propolis-based gel may serve as a valuable adjuvant therapy for cutaneous leishmaniasis [90].

In the next study, mice infected intravenously with *L. infantum* received 500 mg/kg/day of a water extract of Brazilian green propolis orally for 14 days. Treatment reduced liver parasite load by 44%, although spleen burden remained unchanged. Histopathological analyses revealed protection against lesions, including granulomas and vascular alterations. No significant changes were observed in biochemical markers of kidney, liver, or heart function, indicating the safety of the treatment [91].

In another study, mice were subcutaneously infected with *L. major* and treated with a multi-herbal topical preparation containing a mixture of Iranian propolis, *Aloe vera*, *Perovskia abrotanoides*, *Nigella sativa*, lavender, and olive oil. Treatment began four weeks post-inoculation and was applied twice daily. The ointment significantly reduced parasite burden and lesion size, with comparable efficacy to Glucantime, although lesion reduction was somewhat less pronounced [92].

### 5.3. Schistosoma mansonii

In a murine model of *S. mansoni* infection, a dried ethanolic extract of Egyptian propolis was administered orally at a dose of 300 mg/kg daily for four weeks, starting eight weeks post-infection. While propolis alone did not reduce worm burden, it enhanced the antiparasitic effects of praziquantel. Propolis treatment alone significantly decreased hepatic granuloma formation, lymphocytic infiltration, and myeloperoxidase activity in the liver and spleen. It also reduced nitric oxide levels and helped restore plasma protein synthesis and antioxidant status. The authors concluded that propolis acts synergistically with praziquantel and may be a valuable complementary therapy for schistosomiasis [93].

In another study, mice infected with *S. mansoni* were treated with a crude extract of Brazilian red propolis. A single oral dose of 400 mg/kg was administered either 21 days post-infection (early infection) or 49 days post-infection (chronic infection). Propolis treatment led to a significant reduction in the total worm burden—61.26% in early infection and 53.77% in chronic infection. Additionally, the number of immature worm’s eggs in the intestine decreased by 54.71% and 63.11% in early and chronic infections, respectively. A marked reduction in worm’s egg count in feces was also observed—67.3% for early and 59.4% for chronic infections, respectively. The authors concluded that although the reductions were moderate, they were statistically significant, highlighting the potential of Brazilian red propolis as an anthelmintic agent against both early and chronic stages of *S. mansoni* infection [94].

### 5.4. Trypanosoma spp.

In a subsequent study, mice were infected intraperitoneally with *T. cruzi* and treated with Brazilian green propolis ethanolic extract at doses of 25–300 mg/kg/day for 10 days, starting on the first day post-infection. Although no significant changes were observed in parasitaemia curves, propolis-treated mice exhibited delayed mortality by approximately four days compared to untreated controls. No hepatic, renal, or muscular toxicity was observed. The authors suggested that propolis may serve as an adjunctive therapy or as an inhibitor of metacyclogenesis rather than an independent treatment for *T. cruzi* infection [95].

In another study, rats infected intraperitoneally with *T. evansi* received oral ethanolic extract of Brazilian propolis at doses up to 400 mg/kg, starting 24 h post-infection. Although none of the doses were curative, higher doses (300 and 400 mg/kg) prolonged survival (mean longevity of 12.6 and 13.8 days, respectively, versus 6.9 days in controls) and maintained lower parasitaemia for longer durations [96].

### 5.5. Plasmodium spp.

Although the studied *Plasmodium* species do not infect humans, they serve as models in preclinical malaria research. In one study, *P. chabaudi*-infected mice were treated orally with up to 100 mg/kg/day of methanolic Saudi propolis extract for 7 days. Propolis reduced parasitaemia in a dose-dependent manner (49–70%) and significantly ameliorated anaemia, oxidative stress, spleen tissue damage, and immune dysregulation [97].

In another study, *P. berghei*-infected mice were treated 72 h post-infection with intraperitoneal injections of dichloromethane and ethanolic extracts of Iranian propolis (up to 200 mg/kg) for 5 days. Both extracts extended median survival to approximately 26 days compared to 14 days in the control group. Parasitaemia on day 14 was reduced by 65% and 71% for dichloromethane and ethanolic extracts, respectively. However, neither extract prevented mortality, suggesting limited curative efficacy at the tested doses [98].

### 5.6. Other Parasites

An Egyptian research group investigated immunosuppressed rats infected with *Cryptosporidium* spp. Oral treatment with 50 mg/kg of either water or ethanolic extracts of Egyptian propolis was initiated on day 5 post-infection and continued for 7 days. In the first study, the water extract was associated with the lowest mortality (30%) compared to 46% in controls. Propolis also modulated antioxidant enzyme activity and alleviated oxidative stress [99]. In a second study, both extracts significantly reduced oocyst counts (89% for ethanolic and 75% for water extract) by day 7. While they failed to fully restore normal intestinal architecture, they reduced oocyst adhesion to epithelial surfaces and modulated immune responses, indicating therapeutic potential [100].

Furthermore, *Trichinella spiralis* infection was studied in mice treated orally with 250 mg/kg/day of dried ethanolic extract of Egyptian propolis for 35 days, starting one day post-infection. Propolis alone reduced adult worm and muscle larval counts by 75.1% and 77%, respectively. When combined with selenium nanoparticles, reductions were even greater (92.8% and 93.1%). Histopathological evaluation revealed decreased inflammatory infiltration and reduced vascular endothelial growth factor (VEGF) expression in muscles. The authors proposed that this combination may serve as a natural alternative to albendazole for trichinosis treatment [101].

Finally, mice orally infected with *Giardia lamblia* cysts were treated using an aqueous suspension of Biopropolis tablets (1.04 mg/0.2 mL/mouse/day), starting on day 6 post-infection. The results showed a reduction in trophozoite counts from 48% on day 8 to 91% on day 15 post-infection. When combined with olibanum, complete (100%) eradication was achieved. Propolis treatment also improved jejunal histology, decreased inflammation, and eliminated detectable organisms between villi. The authors suggested that propolis combined with olibanum offers a safe, effective alternative to metronidazole, avoiding its known side effects [102]. Overall, propolis reduces parasite burden and granuloma size while promoting protective Th1 responses, suggesting its utility in parasitic disease management. Notably, the combination with conventional drugs such as antimonials or Glucantime enhanced therapeutic efficacy, suggesting a promising role as an adjuvant therapy. The consistency of immune modulation across models supports further investigation in translational settings.

## 6. Antibacterial Effect of Propolis

Bacterial infections remain a major global health concern, particularly in the context of increasing antimicrobial resistance. *Staphylococcus aureus*, including methicillin-resistant strains, is a leading cause of skin, soft tissue, and burn wound infections, often associated with severe clinical outcomes and therapeutic challenges [103]. Gram-negative pathogens such as *Helicobacter pylori*, *Salmonella enterica*, and *Proteus mirabilis* contribute significantly to gastrointestinal, systemic, and urinary tract infections, frequently demonstrating resistance to first-line antibiotics [104,105]. Additionally, polymicrobial infections and biofilm-associated conditions, including those observed in sepsis or dental disease, complicate treatment due to microbial synergy and increased tolerance to conventional therapies [106,107]. The following section reviews experimental studies on the antibacterial activity of propolis against various clinically relevant bacterial species in both mono- and polymicrobial models.

### 6.1. Gram-Positive Bacteria

In the first study, burn wounds on the skin of albino mice were infected with *S. aureus*. A hydroalcoholic extract of South Asian propolis was applied topically to the infected area once daily for 14 days. By day 7, the bacterial load was reduced by 99% in both the 50% and 100% propolis-treated groups, compared to a 45% reduction observed in the untreated control group. By day 14, the burn wound diameter had decreased by 89–98% in the propolis-treated groups, while in the untreated group, the reduction was 65%. At that time, superficial bacterial growth was still detectable in the control group. The authors concluded that propolis represents a promising natural agent for burn wound healing, due to its antibacterial, antioxidant, and anti-inflammatory properties, and noted that it did not cause skin irritation or sensitization [108].

In another study, intradermal infection with *S. aureus* in the ears of mice was treated with Brazilian green propolis. Twenty-four hours after infection, a solution containing 10 µg of propolis was applied topically to the infected ear, either alone or in combination with blue LED light irradiation (450 ± 20 nm), as part of photodynamic therapy (PDT). After 48 h, a significant reduction in bacterial load was observed only in the group receiving PDT. The group treated with propolis alone did not show a statistically significant reduction in bacterial burden. Nevertheless, the authors concluded that propolis exhibited beneficial effects, including prevention of weight loss and cytoprotective activity [109].

### 6.2. Gram-Negative Bacteria

In another study, mice were infected with *H. pylori* and then treated with an ethanolic extract of Korean propolis. The extract was administered orally at a dose of 200 mg/kg, three times per week, over a period of 4 weeks. Negative results in the Campylobacter-like organism test from gastric mucosa samples in the propolis-treated group suggested effective inhibition of *H. pylori* growth. Furthermore, propolis treatment inhibited *H. pylori* virulence factors, reduced pro-inflammatory cytokine production, and alleviated gastric mucosal damage [110].

In a subsequent study, *H. pylori* infection was induced in rats and treated with a hydroalcoholic extract of Brazilian red propolis. The extract was administered orally for 7 days in multiple doses, up to 300 mg/kg. All tested doses significantly reduced the *H. pylori* burden in the antrum (by up to approximately 1 log_10_ CFU/mL), for both the type strain and a clinical isolate. Additionally, the highest dose modulated the local inflammatory response, improving several histological criteria. These included the reduction of chronic inflammation from moderate to mild, the reduction of neutrophilic infiltration from mild to absent levels, and the reduction of tissue damage [111].

*H. pylori* infection in rats was also studied by administering ethanolic extracts of Iranian propolis orally for 21 days at doses up to 300 mg/kg, in combination with probiotic suspensions of *Lactobacillus rhamnosus* and *L. reuteri*. The reduction in bacterial load, assessed via Giemsa staining of gastric tissue, was dose dependent. In addition, propolis treatment was found to ameliorate several histopathological alterations, including erosion depth, haemorrhage, inflammation, and apoptosis levels in the gastric mucosa [112].

In subsequent research, polyurethane ureteric stents were immersed in an ethanolic extract of propolis (128 mg/mL), derived from Heilongjiang Province, China, and incubated at 37 °C for 24 h before drying. The coated stents were implanted into rats, followed by intravesical inoculation with a *P. mirabilis* suspension. The propolis coating significantly reduced both adherent bacteria on the stent surface (approximately threefold) and planktonic bacterial levels in urine (up to 2 log_10_ CFU/mL). Additionally, the coating reduced stone and salt deposition on the stent surface and decreased bladder inflammation, as evaluated seven days after implantation [113].

In another study, mice were infected intraperitoneally with *S. enterica* serovar Typhimurium. The mice were subsequently treated with an ethanolic extract of propolis sourced from Chandigarh, India, administered orally at a dose of 300 mg/kg for 30 consecutive days. In the first study, the treatment resulted in a reduction of bacterial load in the spleen by up to 3 log CFU/g. The authors also noted improvements in haematological parameters, including the amelioration of anaemia, monocytosis, and lymphocytosis, along with normalization of spleen architecture. In contrast, spleens of untreated mice showed marginal zone enlargement, reactive hyperplasia, and increased follicle numbers [114].

In a follow-up study by the same team using a similar protocol, a significant reduction in bacterial load was observed in the blood, liver, spleen, and kidneys—by up to 5 log CFU/g—in propolis-treated mice compared to infected controls. Furthermore, propolis treatment restored liver, kidney, and haematological biochemical markers (including AST, ALT, ALP, bilirubin, urea, uric acid, and creatinine) to near-normal levels. Histological analyses of the liver, spleen, and kidney also revealed normal structural organization and near-normal cytoarchitecture in the treated group, supporting a curative effect of the propolis extract [115].

### 6.3. Polymicrobial Infections

In the study of polymicrobial infection, a standardized extract of Brazilian propolis was administered subcutaneously to mice at doses of 10 mg/kg and 100 mg/kg, six hours prior to the induction of sepsis via the caecal ligation and puncture (CLP) procedure. Although propolis prophylaxis did not reduce bacterial load, it significantly attenuated lung inflammation and decreased the influx of inflammatory cells into the peritoneal cavity. Most notably, the group receiving 100 mg/kg of propolis showed a longer mean survival time, comparable to the antibiotic-treated group. According to the authors, improved survival was primarily attributed to the preservation of lung tissue, with reduced haemorrhage, oedema, inflammation, and necrosis. The authors concluded that propolis may serve as a beneficial adjuvant in prophylactic therapy, particularly in pre-surgical settings, when used alongside antibiotics [116].

In another study, sepsis was induced by CLP in rats, and a hydroalcoholic extract of Brazilian green propolis was administered intraperitoneally at a dose of 500 mg/kg, six hours after the procedure. Although bacterial load was not measured, a significant increase in survival time was observed in the propolis-treated group. Additionally, the treatment protected rats from sepsis-induced acute kidney injury, reduced systemic inflammation and oxidative stress, and preserved the integrity of endothelial cells and mitochondria [117].

Subsequent studies investigated the use of propolis in dentistry, primarily in rat models. In one study, an alcoholic extract of Iranian propolis was formulated into a 3% mouthwash. Rats received 50 mL of the mouthwash orally once daily for two weeks. Using qRT-PCR, researchers evaluated the abundance of four bacterial species constituting the oral microbiota. Compared to chlorhexidine (CHX) and Listerine, propolis mouthwash was more effective in reducing levels of *Streptococcus mutans*, *Enterococcus faecalis*, and *Lactobacillus acidophilus*, and showed similar efficacy to CHX against *S. aureus*. The authors suggested that propolis mouthwash could potentially reduce the risk of periodontal disease, gingivitis, and both primary and secondary oral infections [118].

In another study, an Iranian research group developed a propolis-containing primer (containing up to 10% propolis), which was applied to the labial surface of the maxillary incisors and light-cured. Orthodontic composite was then applied, followed by another layer of the primer, which was also light cured. The rats’ oral cavities were subsequently inoculated with a bacterial suspension containing *S. mutans*, *S. sanguinis*, and *L. acidophilus* for three consecutive days. Bacterial load was assessed on days 1, 4, and 7. The most pronounced reduction of all three bacterial species was observed on day 1. On days 4 and 7, the reduction varied more, but for the 5% and 10% propolis primers, bacterial counts remained lower than in the control group across all species tested [119].

The effectiveness of Egyptian propolis was evaluated in the treatment of periapical pathosis in immature permanent premolars of mongrel dogs. Pulpal exposure was created via endodontic access cavities, followed by the intra-canal application of two propolis-based formulations—a creamy medicament paste and a thicker paste used as a root canal orifice plug. Propolis exhibited antibacterial efficacy comparable to triple antibiotic paste, reducing bacterial counts by approximately 0.9 log_10_ CFU. Moreover, both propolis and mineral trioxide aggregate (MTA) as root canal orifice plugs promoted progressive increases in root length and dentin thickness, along with a reduction in apical diameter. The authors concluded that propolis could be considered a viable substitute for MTA as a root canal orifice plug in regenerative endodontic procedures [120]. Taken together, preclinical studies demonstrate that propolis is especially effective against Gram-positive pathogens, with more variable effects against Gram-negative species. Its ability to reduce bacterial burden and inflammation supports its potential as a complementary antimicrobial therapy. However, findings regarding systemic infections highlight its potential role as an adjunctive agent rather than a stand-alone antimicrobial.

## 7. Antifungal Effect of Propolis

Fungal infections caused by opportunistic pathogens such as *Candida albicans*, *Cryptococcus neoformans*, and *Paracoccidioides brasiliensis* are a growing public health concern, particularly among immunocompromised individuals. *Candida albicans* remains the most common cause of invasive fungal infections worldwide, accounting for high morbidity and mortality, especially in hospital settings. Additionally, the emergence of antifungal resistance highlights the need for alternative therapeutic approaches. Propolis has attracted attention as a natural antifungal agent due to its broad-spectrum activity and favourable safety profile [121,122].

### 7.1. Candida Albicans

Systemic infection with *C. albicans* in mice was treated via oral administration of an ethanolic extract of Iranian propolis. After 18 days of daily treatment at a dose of 100 mg/kg, a significant reduction in fungal burden was observed in the kidneys, brain, and liver compared to the untreated infected group. Moreover, propolis treatment modulated the levels of cytokines such as TNF-α, IFN-γ, and IL-2, although these were measured only in the culture supernatant of splenocytes isolated from the mice [123].

Other studies have investigated vulvovaginal candidiasis (VVC) in BALB/c mice. One such study utilized both aqueous and ethanolic extracts of standardized Brazilian propolis, formulated into 1% mucoadhesive gels. A 60-µL dose was administered intravaginally twice daily, starting three days post-infection. After 10 days of treatment, the fungal burden was reduced by up to 89.4%, comparable to the efficacy of clotrimazole cream. However, the gel also contained essential oils from *Melaleuca alternifolia*, *Betula lenta*, *Mentha spicata*, and *Rosmarinus officinalis*. Histological analysis of vaginal tissue showed preserved tissue architecture and normal epithelial thickness, with no signs of inflammation or *C. albicans* infection in the propolis-treated groups [124].

In a subsequent study by the same research team, new formulations containing 2% or 3% propolis, combined with the same essential oils, were tested. These formulations achieved fungal burden reductions ranging from 83.5% to 95.4% compared to the control group. Notably, the antifungal effect was maintained for an additional 10 days after interruption of treatment. Histopathological evaluation again indicated no signs of irritation or inflammation resulting from the propolis-containing treatments [125].

Another Brazilian research group developed a mucoadhesive thermoresponsive gel incorporating Brazilian propolis extract at concentrations of 14% or 16%. Mice received 60 µL doses intravaginally, twice daily for 7 or 14 days. Both treatment regimens significantly reduced fungal burden—up to a 2 log_10_ CFU/mL reduction after 7 days—with the 16% gel achieving further reduction after 14 days. The antifungal activity was comparable to that of nystatin. Moreover, the 16% formulation was effective against VVC caused by a fluconazole-resistant clinical isolate. Histopathological analysis confirmed the safety of both formulations, showing epithelial tissue without signs of inflammation and with keratinization similar to control animals [126].

The VVC treatment in Wistar rats was investigated using Brazilian green propolis extract encapsulated in poly(ε-caprolactone) nanoparticles coated with hyaluronic acid. The formulation was administered either as a single dose (4.26 mg of extract) to assess antifungal efficacy or daily for 14 days in safety studies. No vaginal toxicity was observed in animals treated with the coated nanoparticles. However, inflammatory infiltration was detected in the mucosa of rats treated with propolis extract alone or with uncoated nanoparticles. Coated nanoparticles demonstrated strong antifungal activity, completely eliminating the fungal burden (a reduction of over 3.5 log_10_ CFU/mL). In contrast, uncoated nanoparticles and propolis extract alone achieved less prominent reductions of approximately 2.8 and 0.6 log_10_ CFU/mL, respectively [127].

### 7.2. Other Fungal Infections

*P. brasiliensis* was subcutaneously injected into an air pouch created in the dorsal region of mice. Beginning 8–10 days post-infection, an ethanolic extract of Brazilian red propolis was administered directly into the air pouch at doses of up to 500 mg/kg. The highest dose produced a strong antifungal effect, significantly reducing the number of viable fungal cells (by 7 log10 CFU/g). Additionally, it led to a decrease in infiltration by neutrophils and an increase in infiltration of monocytes and lymphocytes. Moreover, the treatment reduced ROS production [128].

In a model of systemic infection with *C. neoformans*, mice were treated intravenously with propolis-loaded poly (n-butyl cyanoacrylate) nanoparticles, starting one day after inoculation and continuing until day eight. This treatment significantly reduced the fungal burden in brain tissue (up to 1 log10 CFU/g) and resulted in fewer histopathological lesions compared to the untreated group. Moreover, it also significantly reduced the fungal burden in the lungs and kidneys. The authors concluded that this nanoparticle-based formulation offers a promising targeted approach for treating cerebral cryptococcosis, with the potential to reduce the adverse effects associated with conventional antifungal drugs [129]. Collectively, these findings highlight the antifungal potential of propolis, with consistent reductions in fungal burden and inflammation across different infection sites. However, outcomes were strongly influenced by formulation, with advanced delivery systems (e.g., mucoadhesive gels, nanoparticles) outperforming simple ethanolic extracts. These findings emphasize the need for optimized pharmaceutical formulations to unlock full therapeutic potential.

## 8. Antiviral Effect of Propolis

Viruses from the *Herpesviridae* family, particularly Herpes Simplex Virus types 1 and 2 (HSV-1, HSV-2), are among the most widespread human pathogens. Due to their ability to establish lifelong latency and the growing resistance to conventional antiviral drugs, the search for alternative therapeutic agents remains a major challenge. Recent studies have demonstrated propolis activity against a range of viruses, including DNA viruses such as HSV and RNA viruses like influenza A belonging to the *Orthomyxoviridae* family [130,131].

In a study investigating HSV-1, the skin on the midflank of mice was infected. Starting 4 h before infection, mice received 21 oral doses of a dried ethanolic extract of distinctive Brazilian propolis types (extracts named AF-06, AF-07, and AF-08), dissolved in 1% DMSO, at a dose of 10 mg/kg over a 7-day period. All three tested propolis samples moderately limited the development of skin lesions. Two of the samples (AF-07 and AF-08) significantly reduced the viral titer in the brains and skin of infected mice, and one (AF-08) also showed a significant reduction in the brain (ca. 5 log_10_ PFU/organ). Additionally, two samples enhanced delayed-type hypersensitivity to inactivated HSV-1 antigen, as assessed by footpad swelling. In summary, propolis demonstrated moderate antiviral and immunomodulatory activity [132].

In another study, the scratched mouse skin was infected with HSV-1 and treatment was initiated 4 h post-infection using 8% Brazilian green propolis incorporated into a mucoadhesive thermoresponsive system. Propolis was applied topically five times daily for 10 days. The system was proven to be safe for the skin. Treatment reduced the severity of skin lesions and accelerated healing compared to untreated mice, indicating promise as an antiviral therapy, particularly against acyclovir-resistant HSV-1 strains [133].

A subsequent study evaluated the treatment of intravaginal infection with HSV-2 using a hydroalcoholic extract of brown Brazilian propolis. It was administered orally for five days before and five days after infection. Propolis treatment effectively attenuated extravaginal lesions, prevented lesion progression, and prolonged survival in infected animals. It also reduced inflammation in both the epidermis and dermis while maintaining normal epithelialization, suggesting decreased viral infection. Histopathological analysis revealed no morphological alterations in the propolis-treated group, indicating a lack of observable toxicity at the administered dose (50 mg/kg) [134].

**Figure 2:**
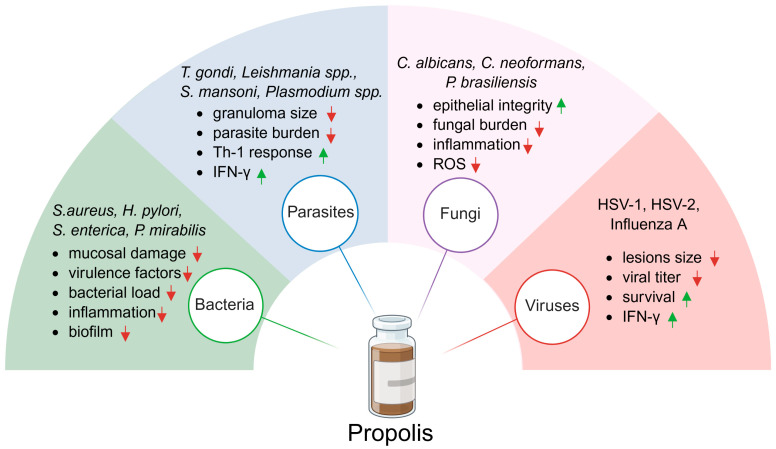
Antimicrobial spectrum of propolis efficacy in vivo. The green arrows indicate an increase or upregulation of the measured parameter, while the red arrows indicate a decrease or downregulation.

In another study, researchers used both aqueous and ethanolic extracts of Brazilian green propolis to treat influenza A virus infection in mice. Propolis was administered orally at a dose of 100 mg/kg, starting 4 h before intranasal inoculation and continued twice daily for six days. Both extracts significantly improved survival compared to the control group, although they did not mitigate the virus-induced weight loss. The suggested mechanism of action involves enhanced viral clearance through increased TNF-related apoptosis-inducing ligand expression [135]. In summary, propolis demonstrates antiviral efficacy against HSV-1, HSV-2, and influenza A, primarily through stimulation of Th1-mediated immunity and enhancement of viral clearance. While lesion size and survival benefits were consistently observed, some outcomes, such as weight loss recovery, showed less improvement. This suggests that antiviral effects may be context-dependent and endpoint-specific. Most of the publications related to propolis activity against the infectious diseases are summarized in Table 3. Furthermore, Figure 2 schematically illustrates the broad antimicrobial spectrum of propolis along with its main biological effects. In summary, propolis reduces bacterial load, virulence factors, inflammation, and biofilm formation in infections caused by, among others, *S. aureus*, *H. pylori*, *S. enterica*, and *P. mirabilis*. It limits parasite burden and granuloma size while enhancing Th1 responses and IFN-γ production in *T. gondii*, *Leishmania spp.*, *S. mansoni*, and *Plasmodium* spp. infections. In fungal infections, including *C. albicans*, *C. neoformans*, or *P. brasiliensis*, propolis decreases fungal burden, inflammation, and ROS levels while preserving epithelial integrity. Finally, in viral infections such as HSV-1, HSV-2, and influenza A, propolis reduces lesions size and viral titers, improves survival, and enhances IFN-γ responses.

## 9. Methods

**Figure 3:**
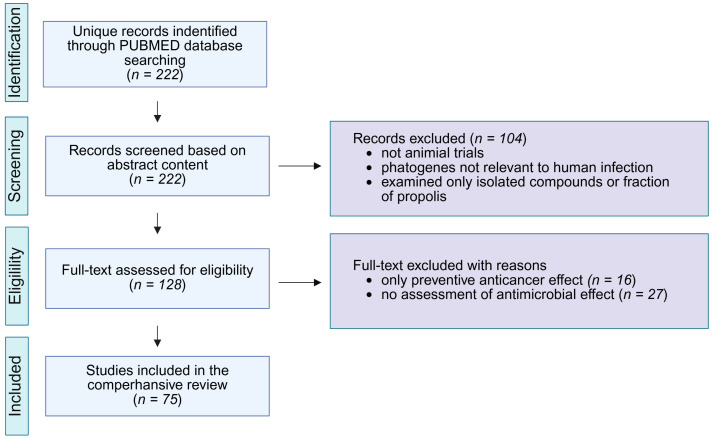
Research methodology for a review process.

A search of the PubMed database was conducted between 27 June and 2 July 2025 (Figure 3). The advanced search tool was used to identify articles containing keywords from three categories in the title and/or abstract: (1) *propolis*; (2) terms indicating the use of an animal model (*mouse*, *mice*, *murine*, *rat*, *animal*, in vivo); and (3) terms related to propolis activity (*bacteria*, *antibacterial*, *parasite*, *antiparasitic*, *virus*, *antiviral*, *yeast*, *fungus*, *antifungal*, *tumor*, *antitumor*, *tumour*, *antitumour*, *cancer*, *anticancer*). The screening was limited to publications from 2010 to 2025. A total of 222 unique records were retrieved, and their abstracts were analyzed. Studies were excluded if they did not involve in vivo experiments, examined only isolated compounds or fractions of propolis without comparison to whole propolis extract, investigated pathogens not relevant to human infection, did not measure the antimicrobial effect of propolis, or focused only on the preventive anticancer effects.

## 10. Perspectives

During the analysis of the reviewed publications, several methodological limitations were observed. In many cases, the geographical origin of the propolis sample was not specified, with the material referred to simply as “propolis”. Additionally, the extraction method, whether aqueous, ethanolic, or otherwise, was often not clearly stated. Furthermore, only a few studies quantified the dry mass content of propolis in their extracts, leading to inconsistencies in dose reporting. Given that the chemical composition of propolis is strongly influenced by its botanical and geographical origin, and that extraction methods significantly affect the nature and concentration of bioactive compounds, the absence of such critical information challenges the interpretation and comparability of results. Another issue concerns the extremely high doses of propolis used in some in vivo experiments, sometimes exceeding 500 mg/kg in mice, which would be impractical for translation to human use. Additionally, several studies administered propolis both before and after tumor inoculation or infection induction, making it impossible to distinguish whether the observed effects were preventive or therapeutic. Notably, the mechanism(s) or mode of action(s), i.e., How propolis works?, especially as an antimicrobial and anticancer agent, should be studied to explain and utilize such results. These limitations highlight the urgent need for standardized reporting guidelines in natural product research. Such protocols should clearly define the essential information to be included in the materials and methods section, particularly regarding source, extraction process, dosage, and timing of administration. Without this information, the reproducibility, translatability, and scientific value of the findings may be significantly compromised.

## 11. Conclusions

In summary, propolis shows consistent anticancer and antimicrobial effects in animal models. Its bioactivity involves apoptosis induction, inhibition of proliferation and angiogenesis, and modulation of immune responses. In infectious models, propolis reduces pathogen burden, alleviates tissue damage, and enhances host defense mechanisms. However, variability in chemical composition, extraction methods, and dosing regimens complicates comparisons across studies and may account for inconsistent results. These limitations highlight the need for standardized preparations and rigorous translational studies before the therapeutic potential of propolis can be reliably evaluated in clinical settings.

## Figures and Tables

**Table 1:** Summary of in vivo studies evaluating the anticancer activity of propolis. The green arrows indicate an increase or upregulation of the measured parameter, while the red arrows indicate a decrease or downregulation.

Cancer	Cell Line	Type of Propolis	Administration	Dose [mg/kg]	Treatment Duration	Effects	Reference
Breast cancer	EAC	Egyptian—water-soluble	oral	50	11 days	⬆ survival ⬇ tumor burden	[30]
Breast cancer	EAC	Egyptian—ethanolic extract	oral	500	2 weeks	⬆ survival ⬇ tumor volume ⬇ methotrexate toxicity	[50]
Cervical cancer	HeLa	Thai—ethanolic extract	oral	24	~1000 mm^3^	⬆ survival ⬆ apoptosis	[34]
Colorectal cancer	HCT-116	Iraq—unknown extract	oral	500, 1000	3 weeks	⬇ tumor volume ⬇ mitosis	[36]
Gastrointestinal cancer	A4gnt KO	Philippine stingless bee—ethanolic extract	oral	100	30 days	⬇ tumor growth ⬇ T-lymphocyte infiltration	[37]
Colorectal cancer	CT26	supercritical CO2 extract of propolis	oral	100–300	1 week	⬇ tumor volume ⬆ immune stimulation	[38]
Epidermoid carcinoma	A431	Poplar—ethanolic extract	oral	50, 100	12 days	⬇ tumor volume ⬆ necrosis	[45]
Fibrosarcoma	HT1080	Brazilian green—supercritical extract	oral	100	3 weeks	⬇ tumor volume ⬇ cell migration	[47]
Hodgkin lymphoma	DLA	Indian—standardized ethanolic extract	i.p.	100–400	14 days	⬆ life span ⬇ tumor growth	[49]
Colorectal cancer	Induced	Iranian—ethanolic extract	i.p.	10–90	8 weeks	⬇ progression ⬆ efficiency of 5-FU	[52]
Colorectal cancer	LoVo-luc	Anatolian—ethanolic extract	i.p./oral	400, 800	3 weeks	⬇ tumor size ⬆ efficiency of 5-FU	[53]
Mammary carcinoma	4T1	Croatian—powder	in food	300	38 days	⬇ tumor size with 5-FU ⬇ metastasis with 5-FU	[54]
Colorectal cancer	Induced	Sericin/propolis nanoparticles	oral	75	8 weeks	⬆ autophagy ⬆ apoptosis ⬇ oxidative stress	[55]
Breast cancer	EAC	Poplar-type—water and ethanolic extracts	i.p.	100	3 days	⬆ survival with Irinotecan ⬆ hematopoietic activity	[57]
Mammary tumor	SMMT	Iranian—ethanolic extract	oral	100	10 days	⬇ tumor size ⬇ Th2 responses ⬆ pro-inflammatory cytokines	[58]
Breast cancer	4T1	Neptune™—water extract	oral	66	14 days	⬇ tumor volume ⬆ immune stimulation	[59]
Epidermoid carcinoma	A431	Brazilian green—ethanolic extract	cells pre-treatment	75 µg/mL	pre-treatment	⬇ tumour volume with PpIX-PDT ⬆ apoptosis with PpIX-PDT	[61]

**Table 2:** Summary of in vivo studies evaluating the toxicity-reducing properties of propolis.

Drug	Dose of Drug [mg/kg]	Type of Propolis	Administration	Dose of Propolis [mg/kg]	Treatment Duration	Main Effect	Reference
5-fluorouracil	125	Irania—ethanolic extract	oral	250	14 days	ameliorates the cardiotoxic effects	[62]
Mitomycin C	8	Indian—hydroethanolic extract	i.p.	100–800	1 h before	mitigates the genotoxic and cytotoxic effects	[63]
Mitomycin C	2–8	Indian—hydroethanolic extract	i.p.	400	1 h before	mitigates testicular damage	[64]
Cisplatin	7	Turkish—water extract	oral	50, 100	14 days	protects against nephrotoxic effects	[68]
Irinotecan	50	Croatian—water and ethanolic extracts	i.p.	100	3 days before	ameliorates haematological, liver, and kidney toxicity	[69]
Doxorubicin	3	Egyptia—ethanolic extract	oral	200	3 weeks	alleviates toxicity to the testis	[70]
Doxorubicin	25	Indian—ethanolic extract	oral	250	14 days	protects against liver toxicity and oxidative stress	[71]
Cyclophosphamide	200	Saudi Arabia—ethanolic extract	i.v.	100	7 days	ameliorates haematological, liver, and kidney toxicity	[72]
Radio-therapy	35 Gy	Anatolian—water-soluble droplets	oral	100, 200	3–7 weeks	reduces the severity of osteoradionecrosis	[77]
Radio-therapy	15 Gy	Turkish—water soluble extract	i.v.	100, 200	2 weeks	reduces acute mucositis	[78]
Radio-therapy	15 Gy	Unknown	i.p.	400	3 days	protects salivary gland function and reduces mucositis	[79]

i.p.—intraperitoneal; i.v.—intravenous.

**Table 3:** Summary of in vivo studies evaluating the activity of propolis against infectious diseases. The green arrows indicate an increase or upregulation of the measured parameter, while the red arrows indicate a decrease or downregulation.

Infectious Agent	Route of Infection	Type of Propolis	Administration	Dose [mg/kg]	Treatment Duration	Effects	Reference
*Toxoplasma gondii*	i.p.	Egyptian—ethanolic extract	oral	150	7 days	⬆ survival ⬇ parasite count	[84]
*Toxoplasma gondii*	i.p.	Egyptian—unknown extract	oral	0.1 mL/day	10 days	⬇ parasite load ⬇ changes in liver and lung	[85]
*Toxoplasma gondii*	i.p.	Egyptian—unknown extract	oral	0.1 mL/day	10 days	⬇ parasite load ⬇ lesions in brain, uterus, kidneys	[86]
*Toxoplasma gondii*	unknown	Unknown	oral	0.1 mL/day	10 days	⬇ parasite load ⬆ histopathology of liver, spleen, lungs	[87]
*Toxoplasma gondii*	i.p.	Egyptian—commercial extract	oral	0.1 mL/day	10 days	⬇ parasite load ⬇ changes in brain, uterus, kidneys	[88]
*Leishmania* *amazonensis*	s.c.	Brazilian Red—ethanolic extract	topical	2.5% gel	20 days	⬇ lesions (combined with Glucantime)	[89]
*Leishmania* *amazonensis*	i.d.	Brazilian Green—glycolic extract	topical	3.6% gel	3 weeks	⬇ lesion size ⬇ inflammation	[90]
*Leishmania* *infantum*	i.v.	Brazilian Green—water extract	oral	500	14 days	⬇ parasite load in liver ⬇ lesions in liver and spleen	[91]
*Leishmania* *major*	s.c.	Iranian in mixture with herbs	topical	twice daily	unknown	⬇ parasite burden ⬇ lesion size	[92]
*Schistosoma mansoni*	s.c.	Egyptian—ethanolic extract	oral	300	4 weeks	⬇ hepatic granuloma number ⬇ lymphocytic infiltration and aggregation ⬆ Praziquantel activity	[93]
*Schistosoma mansoni*	s.c.	Brazilian Red—crude extract	oral	400	single dose	⬇ total worm burden ⬇ eggs number in the intestine and feces	[94]
*Trypanosoma cruzi*	i.p.	Brazilian Green—ethanolic extract	oral	25–300	10 days	⬆ survival ⬇ metacyclogenesis	[95]
*Trypanosoma* *evansi*	i.p.	Brazilian—ethanolic extract	oral	100–400	10 days	⬆ longevity ⬇ parasitaemia levels	[96]
*Plasmodium chabaudi*	i.p.	Saudi—methanolic extract	oral	25–100	7 days	⬇ parasitaemia levels ⬇ oxidative damage ⬆ splenic architecture	[97]
*Plasmodium berghei*	i.p.	Iranian—dichloromethane and ethanolic extracts	i.p.	50–200	5 days	⬆ survival ⬇ parasite count	[98]
*Cryptosporidium* spp.	oral	Egyptian—ethanolic and water extracts	oral	50	7 days	⬇ mortality rate ⬇ oxidative stress	[99]
*Cryptosporidium* spp.	oral	Egyptian—ethanolic and water extracts	oral	50	7 days	⬇ oocysts count ⬆ total leukocytic count	[100]
*Trichinella* *spiralis*	oral	Egyptian—ethanolic extract	oral	250	34 days	⬇ worm and larval count ⬇ inflammation and angiogenesis	[101]
*Giardia lamblia*	oral	‘Biopropolis‘ tablets—aqueous suspension	oral	1.04 mg/ 0.2 mL/mouse	7 days	⬇ trophozoite count ⬇ changes in jejunal mucosa	[102]
*Staphylococcus aureus*	topical	South Asian—hydroalcoholic extract	topical	50, 100%	13 days	⬇ wound diameter ⬇ bacterial load	[108]
*Staphylococcus aureus*	i.d.	Brazilian Green—glycolic extract	topical	10 µg	single dose	⬇ bacterial load (combined with aPDT) ⬇ weight loss	[109]
*Helicobacter* *pylori*	oral	Korean—ethanolic extract	oral	200	4 weeks	⬇ bacterial growth and virulence factors ⬇ lesions and inflammation	[110]
*Helicobacter* *pylori*	oral	Brazilian Red—hydroalcoholic extract	oral	18–300	7 days	⬇ bacterial load ⬇ chronic inflammation	[111]
*Helicobacter* *pylori*	oral	Iranian—ethanolic extract	oral	75–300	21 days	⬇ bacterial load ⬇ changes in gastric tissue	[112]
*Proteus mirabilis*	intravesical	Chinese—ethanolic extract	coating on ureteric stents	128 mg/mL	prevention	⬇ bacteria, stones, salt deposits ⬇ inflammation	[113]
*Salmonella* *enterica* serovar Typhimurium	i.p.	Indian—ethanolic extract	oral	100, 300	30 days	⬇ bacterial load ⬇ biochemical and histological changes	[114]
*Salmonella* *enterica* serovar Typhimurium	i.p.	Indian—ethanolic extract	oral	300	30 days	⬇ bacterial count ⬆ biochemical, haematological parameters	[115]
Polymicrobial-sepsis	CLP	Brazilian—standardized extract	s.c.	10, 100	single dose	⬆ survival ⬇ lung inflammation	[116]
Polymicrobial-sepsis	CLP	Brazilian Green—ethanolic extract	i.p.	500	single dose	⬆ survival ⬇ acute kidney and lung injury	[117]
Cariogenic bacteria	–	Iranian—ethanolic extract	oral	3 mg	2 weeks	⬇ bacterial count	[118]
Cariogenic bacteria	oral	Iranian—nanoparticles	tooth surface	1–10%	single dose	⬇ bacterial count	[119]
Polymicrobialteeth	–	Egyptian—unknown extract	tooth canal	150	single dose	⬇ bacterial count ⬆ teeth regeneration	[120]
*Candida albicans*	i.v.	Iranian—ethanolic extract	oral	100	18 days	⬇ fungal burden ⬇ proinflammatory cytokines	[123]
*Candida albicans*	i.vag.	Brazilian—ethanolic and water extracts	i.vag.	1% 60 µL	10 days	⬇ fungal burden	[124]
*Candida albicans*	i.vag.	Brazilian—standardized extract	i.vag.	2–3%	10 days	⬇ fungal burden ⬇ changes in vaginal tissue	[125]
*Candida albicans*	i.vag.	Brazilian—ethanolic extract	i.vag.	14–16% 60 µL	14 days	⬇ fungal burden	[126]
*Candida albicans*	i.vag.	Brazilian Green—nanoparticles	i.vag.	4.26 mg	24 h	⬇ fungal burden (full composition)	[127]
*Paracoccidioides brasiliensis*	s.c.	Brazilian Red—ethanolic extract	s.c.	50–500	single dose	⬇ fungal burden ⬆ neutrophils activity	[128]
*Cryptococcus neoformans*	i.v.	Thai—nanoparticles	i.v.	30.75 mg	8 days	⬇ fungal burden in brain ⬇ lesions number in brain	[129]
*Herpes Simplex*—Type 1	s.c.	Brazilian—ethanolic extracts	oral	10	6 days	⬇ virus titers ⬇ skin lesions	[132]
*Herpes Simplex*—Type 1	topical	Brazilian Green—unknown extract	topical	8%	10 days	⬇ lesion score	[133]
*Herpes Simplex*—Type 2	i.vag.	Brazilian Brown—hydroalcoholic extract	oral	50	5 days	⬇ lesion score ⬆ longevity ⬇ inflammation	[134]
*Influenza A* virus	intranasal	Brazilian Green—ethanolic and water extracts	oral	100	6 days	⬆ survival ⬆ viral clearance	[135]

i.p.—intraperitoneal; s.c.—subcutaneous; i.d.—intradermal; i.v.—intravenous; i.vag.—intravaginal.

## Data Availability

Not applicable.
